# Frequency and Intensive Care Related Risk Factors of Pneumothorax in Ventilated Neonates

**DOI:** 10.1155/2014/727323

**Published:** 2014-04-28

**Authors:** Ramesh Bhat Yellanthoor, Vidya Ramdas

**Affiliations:** Department of Paediatrics, Kasturba Medical College, Manipal University, Manipal, Udupi District, Karnataka 576104, India

## Abstract

*Objectives*. Relationships of mechanical ventilation to pneumothorax in neonates and care procedures in particular are rarely studied. We aimed to evaluate the relationship of selected ventilator variables and risk events to pneumothorax. *Methods*. Pneumothorax was defined as accumulation of air in pleural cavity as confirmed by chest radiograph. Relationship of ventilator mode, selected settings, and risk procedures prior to detection of pneumothorax was studied using matched controls. *Results*. Of 540 neonates receiving mechanical ventilation, 10 (1.85%) were found to have pneumothorax. Respiratory distress syndrome, meconium aspiration syndrome, and pneumonia were the underlying lung pathology. Pneumothorax mostly (80%) occurred within 48 hours of life. Among ventilated neonates, significantly higher percentage with pneumothorax received mandatory ventilation than controls (70% versus 20%; *P* < 0.01). Peak inspiratory pressure >20 cm H_2_O and overventilation were not significantly associated with pneumothorax. More cases than controls underwent care procedures in the preceding 3 hours of pneumothorax event. Mean airway pressure change (*P* = 0.052) and endotracheal suctioning (*P* = 0.05) were not significantly associated with pneumothorax. Reintubation (*P* = 0.003), and bagging (*P* = 0.015) were significantly associated with pneumothorax. *Conclusion*. Pneumothorax among ventilated neonates occurred at low frequency. Mandatory ventilation and selected care procedures in the preceding 3 hours had significant association.

## 1. Introduction

Pneumothorax is the most common air-leak syndrome resulting in significant morbidity and mortality in neonates [1–3]. Increased mortality and chronic lung disease with pneumothorax (about 13 times) in very low birth weight (VLBW) neonates have been reported by Powers and Clemens [3]. Its relationship with underlying primary lung disorders is well recognized [1, 4, 5]. However, higher incidences of pneumothorax in ventilated neonates [3–5] with mild increase among those receiving continuous positive airway pressure (CPAP) and dramatic increase with mandatory modes of ventilation have been addressed by only few studies [1, 4]. Similarly studies addressing association of pneumothorax to various ventilation strategies are scarce. Such strategies with high incidence of pneumothorax include high peak inspiratory pressure (PIP) and mean airway pressure (MAP), active expiratory reflex, administration of bag and mask ventilation, endotracheal tube displacement, an increase in clinical interventions [6–9], long inspiratory time [10], and high frequency ventilation [11]. Ventilator rates greater than 60/min associated with decreased risk of pneumothorax were found by Greenough et al. [9]. Maximal PIP and suction have been found to be risk factors for pneumothorax in a study by Klinger et al. [4]. A mortality of 10% to 38.6% with neonatal pneumothorax has been reported by various studies [12, 13]. Whether associated variables are causative or merely a result of an undiagnosed air leak is debatable. Studies in this regard can help define care practices or risk factors associated with pneumothorax. We aimed to study the association of ventilator mode, selected settings, and care procedures with the development of pneumothorax in ventilated neonates.

## 2. Methods

Newborns who were detected to have pneumothorax while receiving mechanical ventilation between May 2006 and August 2008 were studied. Clinically suspected pneumothorax was subjected to transillumination test using cold light (Karl Storz endoscopy) and chest X-ray. Pneumothorax was defined as accumulation of air in the pleural cavity as confirmed by chest radiograph. To study the association of ventilator parameters and other potential risk events with the development of pneumothorax among the ventilated neonates, birth weight and gestational age matched controls were selected [6]. The subsequent two neonates who were ventilated following each case without developing an air leak and gestation within two weeks and birth weight within 500 g of the cases constituted the controls. A PaCO_2_ < 30 mm Hg was considered as an indicator of overventilation. To assess this, the minimum PaCO_2_ for each case in the preceding 3–6 hours of the diagnosis of pneumothorax and the minimum PaCO_2_ of the matched control before that same age were noted. Ventilatory details and arterial blood gases were retrieved up to the age of diagnosis in the cases. For the preceding three hours, the data regarding procedures such as endotracheal suction, reintubation, and bagging were retrieved from the neonatal case records. The three-hour period was chosen similar to the previous study [6]. Newborns were ventilated using Drager 8000 Neonatal Ventilator (Drager Ltd, Hemel Hempstead, UK), Bear Cub Infant Ventilator, or Infant Star Ventilator. Mode of ventilation and sedation with fentanyl were selected as per clinician's choice. The initial ventilation mode was intermittent mandatory ventilation/assist control (IMV/AC); subsequently it was changed to synchronised IMV (SIMV)/CPAP as guided by arterial blood gas analysis and clinical improvement. The sedation involved infusion of fentanyl, 2–4 microgram/kg/hour. Surfactant was used as an early rescue therapy only. We did not use neuromuscular blockade in any case. Any increase in the mean airway pressure (MAP) in the preceding 3 hours of pneumothorax was considered as MAP change. Ethical approval for the study from the hospital ethical committee and informed consents from parents were obtained. Data was analysed using Statistical Package for Social Sciences (SPSS) software version 11.5. Proportions were compared and analyzed using Fischer's exact test or chi-square test.

## 3. Results

During the study period, 10 (1.85%) neonates had pneumothorax out of 540 who received mechanical ventilation. Mean (SD) birth weight of cases was 2250 (450) g and controls were 2150 (530) g. Birth weight range was 775–3820 g among cases and 840 g–4000 g among controls. Median gestational age of cases was 36 weeks (range, 26–40 weeks) and controls 36 weeks (range 28–40 weeks). Mode of delivery by vaginal route and cesarean section were not significantly different among cases and controls. Six among cases and 10 among controls received mask or tube ventilation at birth. Among cases, 6 had hyaline membrane disease (HMD), 3 had meconium aspiration syndrome (MAS), and 1 had pneumonia. Among controls, 14 had HMD and 6 had MAS. Most of the pneumothoraces (80%) were diagnosed within the first 48 hours of life. Three pneumothoraces among infants with HMD and two among infants with MAS were diagnosed between 24 and 72 hours. Of 10 neonates with pneumothorax receiving mechanical ventilation, 5 died. The mortality among ventilated neonates with pneumothorax was nearly twice that of neonates without pneumothorax (50% versus 26.7%).

Table 1 shows relationship of selected ventilator variables and overventilation to pneumothorax. Among neonates with pneumothorax who were on ventilator, 70% were on mandatory positive pressure ventilation as compared to 20% among controls. Pneumothorax event had significant association (*P* < 0.01) with mandatory positive pressure ventilation as compared to other modes. Peak inspiratory pressure (PIP) > 20 cm H_2_O or overventilation as evidenced by PaCO_2_ <30 mmHg was not significantly (*P* = 0.58 and 0.79, resp.) associated with occurrence of pneumothoraces.

Table 2 shows the relationship of care procedures in the preceding 3 hours of pneumothorax event. Endotracheal suctioning and mean airway pressure (MAP) change were observed in higher proportions among cases as compared to controls. However, these differences were not statistically significant. Reintubation and bagging had significant association (*P* < 0.05) with occurrence of pneumothorax.

## 4. Discussion

Pneumothorax in newborn results in significant morbidity and mortality [1–5, 14]. It may even increase chronic lung disease in VLBW neonates [3] and intraventricular hemorrhage in preterm neonates [12]. Hence pneumothorax warrants preventive measures. Mechanical ventilation is said to increase the pneumothorax incidence in neonates and such incidence varies widely. Watkinson and Tiron [6] reported that 8.7% ventilated neonates developed at least one pneumothorax during the first two weeks of life. A higher incidence of 10%–13.4% has been reported by studies from UK [15, 16] and of 26% by Malek et al. from Iran [17]. We observed a low pneumothorax incidence of 1.85% among ventilated neonates. However, this low incidence refers to pooled neonates of low and normal body weight together and symptomatic pneumothoraces only. Morley et al. [18] reported incidence of pneumothorax as 9% after CPAP. In a case-control study on VLBW infants from 1997 to 2002, pneumothorax rate was 10.9% [4].

Improvement in mechanical ventilator strategies to reduce pulmonary complications and improve long-term outcomes has been debated. Pneumothorax event, in the present study, had significant association with mandatory positive pressure ventilation than other modes. Different rates of pneumothorax with different modes of ventilation are recognised. Pneumothorax rate of about 16% among those receiving CPAP and 34% for those being ventilated was observed by Greenough and Millner [5]. An analysis of 16 trials comparing elective high-frequency ventilation with conventional ventilation [11] showed a significant increase in air leakage in the high-frequency group (29% versus 24%).

Although higher percentage of newborns among cases had PIP >20 cm H_2_O than control group in the present study, this difference was not statistically significant. This is similar to a previous observation [6]. In contrast, a study on VLBW infants found maximal PIP during the 24 hours before diagnosis of pneumothorax as significant risk factor for pneumothorax [4]. Watkinson and Tiron [6] in their analysis of 606 ventilated neonates reported that overventilation defined as a PaCO_2_ <30 mm Hg was not associated with pneumothorax. Our study agrees with this report. These findings suggest the need for further studies to resolve the controversial association of higher PIP with occurrence of pneumothorax in ventilated neonates.

A number of care procedures in neonates receiving ventilation associated with pneumothorax have been reported [6–8, 19]. They include an increase in clinical interventions such as suction procedures, chest radiography, reintubation, chest compressions, and active expiration. The number of suction procedures during the 8 hours before diagnosis of pneumothorax with an odds ratio of 1.56 (95% CI, 1.09–2.23) was previously reported [4]. McIntosh et al. [20] reported that in most cases endotracheal tube was aspirated and at least 40% of newborns were reintubated before the diagnosis of pneumothorax. On the other hand, Primhak had found that slightly elevated PIP and MAP had association with pneumothorax but finally it was longer inspiratory time which was the major risk factor responsible for pneumothorax [21]. In the present study, reintubation and bagging in the preceding three hours were significantly associated with pneumothorax. Higher percentage of cases had MAP change than controls but it was not statistically significant. Together, these findings suggest the need for more supervision and monitoring of these potential risk procedures.

The mortality among ventilated newborns with pneumothorax was nearly twice that of neonates without pneumothorax. This increased mortality is at least partly related to pneumothorax than solely to comorbidities. Comorbidities included persistent pulmonary hypertension (PPHN) in two and one case each of sepsis, pulmonary hemorrhage, and intraventricular hemorrhage. It is likely that pneumothorax had some association with PPHN and IVH also. Identifying risk factors for pneumothorax in ventilated neonates may reduce mortality and improve long-term outcome among survivors if subsequent air leak is prevented. The findings of the present study also positively reinforce an earlier statement that an apparent need to reintubate or bagging procedures in ventilated neonates must be accompanied by a prompt search for a pneumothorax [6].

The present study had certain limitations. Firstly, it was a single centre study. The study samples were small due to low incidence of pneumothorax although a large number of neonates received mechanical ventilation. Secondly, the adequacy of sedation among study population was monitored only clinically and not objectively by any sedation score.

In conclusion, the incidence of pneumothorax among ventilated neonates in the present study is much lower than previous studies. High mortality of pneumothorax among neonates receiving mechanical ventilation remains as a cause of concern. Regardless of whether associated variables are causative or merely a result of an undiagnosed air leak, more attention to these potential care procedures is warranted.

## Figures and Tables

**Table 1:** Relationship of ventilator variables and overventilation to pneumothorax.

Parameter	Cases (*n* = 10)		Controls (*n* = 20)		*P* value
	*n*	%	*n*	%	
Mode of ventilation at pneumothorax event					
IMV	7	70	4	20	0.007
AC	2	20	4	20	
SIMV	0	0	4	20	
CPAP	1	10	8	40	
PIP >20 cm H_2_O	4	40	6	30	0.58
PaCO_2_ <30 mmHg in ABG	5	50	11	55	0.79

IMV: intermittent mandatory ventilation; AC: assist control; SIMV: synchronized intermittent mandatory ventilation; CPAP: continuous positive airway pressure; PIP: peak inspiratory pressure; ABG: arterial blood gas.

**Table 2:** Relationship of risk events to pneumothorax.

Risk events	Cases (*n* = 10)		Controls (*n* = 20)		OR	95% CI	*P* value
	*n*	%	*n*	%			
Endotracheal suctioning	6	60	6	30	3.5	0.7–17.1	0.05
Mean airway pressure change	4	40	2	10	6.0	0.8–41.4	0.052
Reintubation	6	60	2	10	13.5	1.9–93.2	0.003
Bagging	5	50	2	10	9.0	1.3–61.1	0.015

OR: odds ratio; CI: confidence interval.
